# Regulation Effects of Water and Nitrogen on the Source-Sink Relationship in Potato during the Tuber Bulking Stage

**DOI:** 10.1371/journal.pone.0146877

**Published:** 2016-01-11

**Authors:** Wenting Li, Binglin Xiong, Shiwen Wang, Xiping Deng, Lina Yin, Hongbing Li

**Affiliations:** 1 Stata Key Laboratory of Soil Erosion and Dryland Farming on the Loess Plateau, Institute of Soil and Water Conservation, Chinese Academy of Sciences / Northwest A&F University, Yangling, Shaanxi, China; 2 University of Chinese Academy of Sciences, Beijing, China; Institute for Sustainable Plant Protection, C.N.R., ITALY

## Abstract

The source-sink relationship determines crop yield, and it is largely regulated by water and nutrients in agricultural production. This has been widely investigated in cereals, but fewer studies have been conducted in root and tuber crops such as potato (*Solanum tuberosum* L.). The objective of this study was to investigate the source-sink relationship in potato and the regulation of water and nitrogen on the source-sink relationship during the tuber bulking stage. A pot experiment using virus-free plantlets of the Atlantic potato cultivar was conducted, using three water levels (50%, 70% and 90% of field capacity) and three nitrogen levels (0, 0.2, 0.4 g N∙kg^−1^ soil). The results showed that, under all water and nitrogen levels, plant source capacity were small at the end of the experiment, since photosynthetic activity in leaves were low and non-structural reserves in underground stems were completely remobilized. While at this time, there were very big differences in maximum and minimum tuber number and tuber weight, indicating that the sink tuber still had a large potential capacity to take in assimilates. These results suggest that the source-supplied assimilates were not sufficient enough to meet the demands of sink growth. Thus, we concluded that, unlike cereals, potato yield is more likely to be source-limited than sink-limited during the tuber bulking stage. Water and nitrogen are two key factors in potato production management. Our results showed that water level, nitrogen level and the interaction between water and nitrogen influence potato yield mainly through affecting source capacity via the net photosynthetic rate, total leaf area and leaf life span. Well-watered, sufficient nitrogen and well-watered combined with sufficient nitrogen increased yield mainly by enhancing the source capacity. Therefore, this suggests that increasing source capacity is more crucial to improve potato yield.

## Introduction

Expanding population and consumption growth will increase the global agricultural productivity need before 2050 [1, 2]. This increase should be achieved without additional farming land and inputs [3]. Thus, crop yield improvement is needed to produce higher yields on a per-plant basis with fewer inputs [4]. Understanding the determinate factors in per-plant yield is important for crop yield improvement. Generally, the organ or site in crop plants that synthesizes assimilates is called a source, such as the mature leaf. The site where synthesized assimilates accumulate is known as a sink [5]. Crop yield, represented by the harvested organs, is influenced by the production of assimilates by the source and the extent to which they can accumulate in the sink [6]. If the sink is small, the yield cannot be high; however, if the sink is large, the yield also cannot be high if the source capacity is limited [5]. For example, in wheat, many studies suggest that the grain yield is sink-limited during the grain filling stage, so increasing the sink capacity would increase the yield potential [7–10]. Therefore, determining whether the source or sink limits yield is critical for increasing crop yield potential.

However, the source-sink relationship for yield in crops is not static and is influenced by both genotype and external environmental factors [11]. Variation of environmental factors is the major cause for different yields [12]. While many of the environmental factors can be modulated by farmers, water and nitrogen are the main factors that control plant growth [13]. Water is essential for plant growth and its deficiency is one of the most important factors limiting crop yield [14, 15]. Previous studies in cereals showed that water deficiency could induce large alterations in the source-sink relationship because of a modification of growth priorities, such as decreasing photosynthetic rate and grain size and number, which results in yield fluctuations [16–18]. Nitrogen is also an important element that affects crop yield [19]. Warraich et al. concluded that applied nitrogen fertilizer could improve wheat yield by improving both source and sink efficiency by increasing the leaf area index, relative growth rate, net assimilation rate, grain filling rate and grain filling duration [20]. In addition, nitrogen also significantly affects crop yield under water deficient conditions. Madani et al. showed that in winter wheat under water deficient conditions, supplying post-anthesis nitrogen fertilizer increases grain yield by decreasing the sink limitation, and not by increasing source strength [21]. Therefore, investigating the source-sink relationship in crops and its effects on yield under different water and nitrogen conditions helps to maximize crop yield and optimize water and nitrogen use efficiency.

Potato (*Solanum tuberosum* L.) is an important crop with more than 368 million tons of tubers produced on all continents in 2013. It ranks as the fourth largest global food crop only after maize, rice and wheat, and over 1 billion people eat potato as their staple diet (http://faostat.fao.org). Potato yield significantly affects global food security [22]. Thus, understanding the source-sink relationship in potato is important for improving its tuber yield and for food security. In addition, potato is a tuber crop and the part that is harvested is a type of stem modification. Thus, it may be a suitable model plant to study the source-sink relationship in tuber crops. In cereals such as wheat and rice, the source-sink relationship has been widely investigated, but studies in potato are fewer. Therefore, investigation of the source-sink relationship in potato is important to understand yield improvement in tuber crops. Water and nitrogen also are two main factors that affect potato yield in agricultural production. Previous studies showed that, compared to other crops, potato is more sensitive to water stress, especially during the tuber bulking period [23, 24]. Water stress could decrease the photosynthetic rate, leaf area, tuber number per stem and average tuber weight, thereby reducing the yield [25–29]. Nitrogen could influence leaf area, active life span, chlorophyll content, tuber size and tuber bulking time to affect yield [30–32]. In addition, the combined effect of water and nitrogen on potato yield also has been intensively studied, but most studies have focused on levels that can achieve the highest yield [33–38]. How water and nitrogen regulate the source-sink relationship and how the change of source-sink relationship further affect yield are not clear. Therefore, investigation of the two underling mechanisms is useful for improving potato yield and water/nitrogen use efficiency.

The objective of this study was to analyze the source-sink relationship in potato and its regulation of yield using different water and nitrogen levels. We also conducted our investigation with the following goals: (1) to determine the source-sink relationship in potato regulated by different water and nitrogen levels (2) to access the effect of these different source-sink relationship on yield and (3) to compare potato’s source-sink relationship with that of cereals.

## Materials and Methods

### Growth conditions, plant materials, water and nitrogen treatment

The experiment was performed in Yangling, Shaanxi Province, China (34°12′N, 108°7′E, altitude 530 m). Virus-free plantlets of the potato cultivar Atlantic were propagated in 90 mm Petri dishes containing Murashige and Skoog (MS) medium, supplemented with 2% sucrose and 0.8% agar at 20 ± 2°C, with 14 h light and a light intensity 300 μmol m^−2^ s^−1^ in a growth chamber. Four weeks after subculture, plantlets were transferred to 7.5 cm diameter paper cups containing compost and growth continued in this growth chamber. After growth for 4 weeks in paper cups, on 11 April, 2014, plantlets of uniform height (approximately 20 cm) were transplanted into 21.2 L plastic pots, filled with 9.5 kg air-dried loessal soil. The loessal soil was collected from fields at Ansai Research Station of Chinese Academy of Sciences (36°51′N, 109°19′E, altitude 1,068–1,309 m) located on the Loess Plateau. The soil was Calcic Cambisols according to the FAO/UNESCO Soil Classification. Its pH value was 8.27, organic matter content was 2.4 g∙kg^−1^, and total nitrogen (N), phosphorous (P) and potassium (K) contents were 0.32, 0.68 and 19.6 g∙kg^−1^, respectively. Nitrate-N, Ammonium-N, Olsen-P and available K contents were 16.91, 38.72, 24.82 and 276.13 mg∙kg^−1^, respectively. Soil collection was permitted by the owner of the fields and we confirm that it did not involve endangered or protected species. There was one seedling per pot, and the pots were placed in a field under a transparent rainfall shelter to exclude natural precipitation. Soil gravimetric water content was maintained at 90% of field capacity in all pots by adding sufficient water at 18:00 h every day until the beginning of tuber bulking.

**Fig 1:**
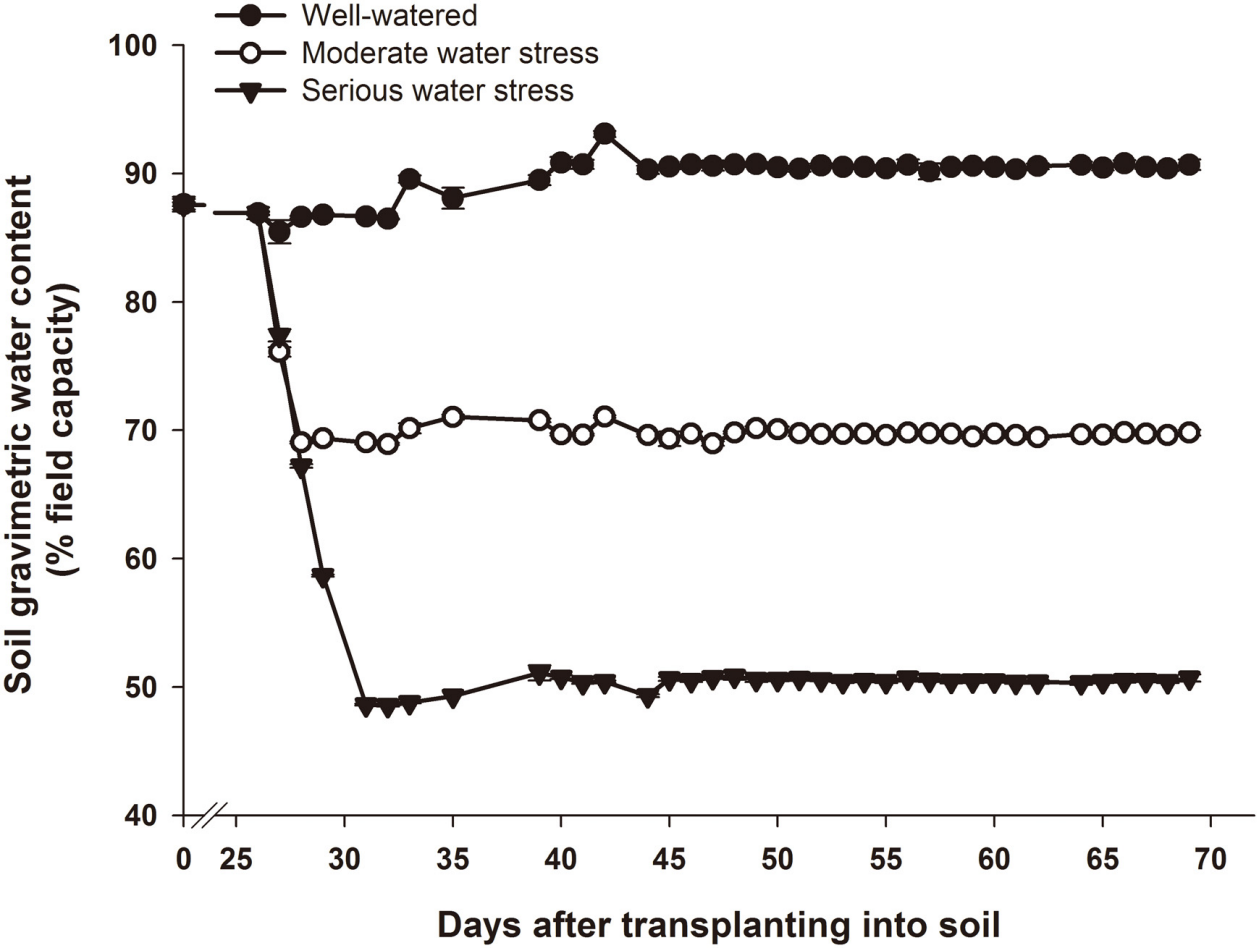
Changes in soil water content during the experiment. Data are presented as the mean ± SE (n ≥ 27).

The treatment consisted of 3 water levels and 3 nitrogen levels (Table 1). The three nitrogen levels were designated as deficient (no N added), sufficient to give a maximum yield and excess (exceeding crop needs), based on our preliminary experiment. The levels were 0, 0.2 and 0.4 g pure nitrogen (N) as urea per kilogram soil, respectively (equivalent to 0, 85.5 and 171 kg N ha^−1^, respectively, since potato generally are planted at a density of 45,000 plants ha^−1^). Phosphate (0.2 g P_2_O_5_ as Ca(H_2_PO_4_)_2_ per kilogram soil, equivalent to 85.5 kg P_2_O_5_ ha^−1^) and potassium (0.1 g K_2_O as K_2_SO_4_ per kilogram soil, equivalent to 42.8 kg K_2_O ha^−1^) fertilizers were applied equally to each treatment. The nitrogen, phosphate and potassium fertilizers were applied into soil before transplanting. The beginning of tuber bulking was observed at 26 days after transplanting into soil (DAT). Water levels were then imposed by withholding watering based on weighting pots every day. For the well-watered group, soil gravimetric water content was maintained at 90% of field capacity. For the moderate water stress group, soil gravimetric water content was maintained at 70%, and for the serious water stress group, it was maintained at 50%. The change of soil gravimetric water content during the experiment was shown in Fig 1. There were 45 pots for each treatment, and different treatments were displayed in a randomized complete block design. At 69 DAT, the experiment ended.

**Table 1:** Water and nitrogen levels in different treatments.

Water	Nitrogen
	Deficient nitrogen (0 g N∙kg^−1^ added to soil)
Well-watered (90% soil water content)	Sufficient nitrogen (0.2 g N∙kg^−1^ added to soil)
	Excess nitrogen (0.4 g N∙kg^−1^ added to soil)
	Deficient nitrogen (0 g N∙kg^−1^ added to soil)
Moderate water stress (70% soil water content)	Sufficient nitrogen (0.2 g N∙kg^−1^ added to soil)
	Excess nitrogen (0.4 g N∙kg^−1^ added to soil)
	Deficient nitrogen (0 g N∙kg^−1^ added to soil)
Serious water stress (50% soil water content)	Sufficient nitrogen (0.2 g N∙kg^−1^ added to soil)
	Excess nitrogen (0.4 g N∙kg^−1^ added to soil)

Eight plants per treatment were sampled at 26, 36, 47, 58 and 69 DAT. Aboveground parts were separated into leaves and aboveground stems. Underground parts were washed to clean the soil residue and were separated into underground stems, roots and tubers. Tubers with the peel were sliced into thin slices. Each part of the plant was firstly dried at 105°C for 30 min and then at 80°C to a constant weight according to Sun et al. [39].

### Biomass measurements

Aboveground biomass was calculated as the sum of the dry weight of leaves and aboveground stems. Tubers larger than 1 cm in diameter were counted and weighed according to Jefferies and Mackerron [40] and Deblonde and Ledent [25]. Tuber yield was calculated as the tuber fresh weight per plant (g/plant). The average tuber weight was the ratio of tuber yield to tuber number.

### Photosynthetic rate

The photosynthetic rates were measured between 9:00 and 11:00 a.m. at 26, 47 and 69 DAT using a portable photosynthesis system (Li-6400, LI-COR Inc., Lincoln, NE, USA) according to Basu et al. [26]. The latest fully expanded leaf was placed in the chamber at a photon flux density of 1000 μmol m^−2^ s^−1^, and the flow rate through the chamber was 500 μmol s^−1^. At 36 and 58 DAT, the photosynthetic rates were not measured because it rained on these days.

### Total leaf area

Total leaf area was calculated as the sum of all leaf areas per plant, which was determined using a scanner (Epson Perfection V700 Photo, Seiko Epson Corporation, Beijing, China) and analyzed with WinRHIZO PRO 2009 software (Regent Inc., Quebec, Canada) according to Liu et al. [41].

### Non-structural reserves content

Dried leaf and underground stem samples were ground using a Retsch MM400 mixer mill (Retsch GmbH, Haan, Germany) and passed through a 40 mesh screen, then their total soluble sugar content was determined by sequential ethanol extractions followed the anthrone method [42]. Starch was extracted from the ethanol-insoluble residue and measured using the anthrone reagent, according to Clegg [43]. Non-structural reserves content was calculated as the sum content of soluble sugar and starch.

### Nitrogen content

Finely ground dried leaf, stem (aboveground + underground) and tuber samples (approximately 0.1 g) were digested in sulfuric acid, with 0.23 g K_2_SO_4_ and 0.07 g CuSO_4_ as catalysts. Nitrogen content was determined by the standard macro-Kjeldahl method according to Nelson and Sommers [44] using a Kjeltec 2300 Analyzer Unit (Foss Tecator AB, Hoganas, Sweden).

### Statistical analysis

Data populations obtained from the experiment had demonstrable normal distributions. A two-way analysis of variance (ANOVA) was used to assess the effects of water level, nitrogen level, and their interaction on all dependent variables. The normal distribution was tested using the Levene’s Test. Analysis of variance (ANOVA) was performed using SPSS statistical software (Version 19.0 for Windows, SPSS, Chicago, USA). A General Liner Model analysis of variance in the SPSS System was adopted. Water level and nitrogen level were both the fixed effect. Pearson correlation analyses in the SPSS System were used to assess correlations between tuber yield and other relevant parameters.

## Results

### Effects of different water and nitrogen levels on tuber yield

**Fig 2:**
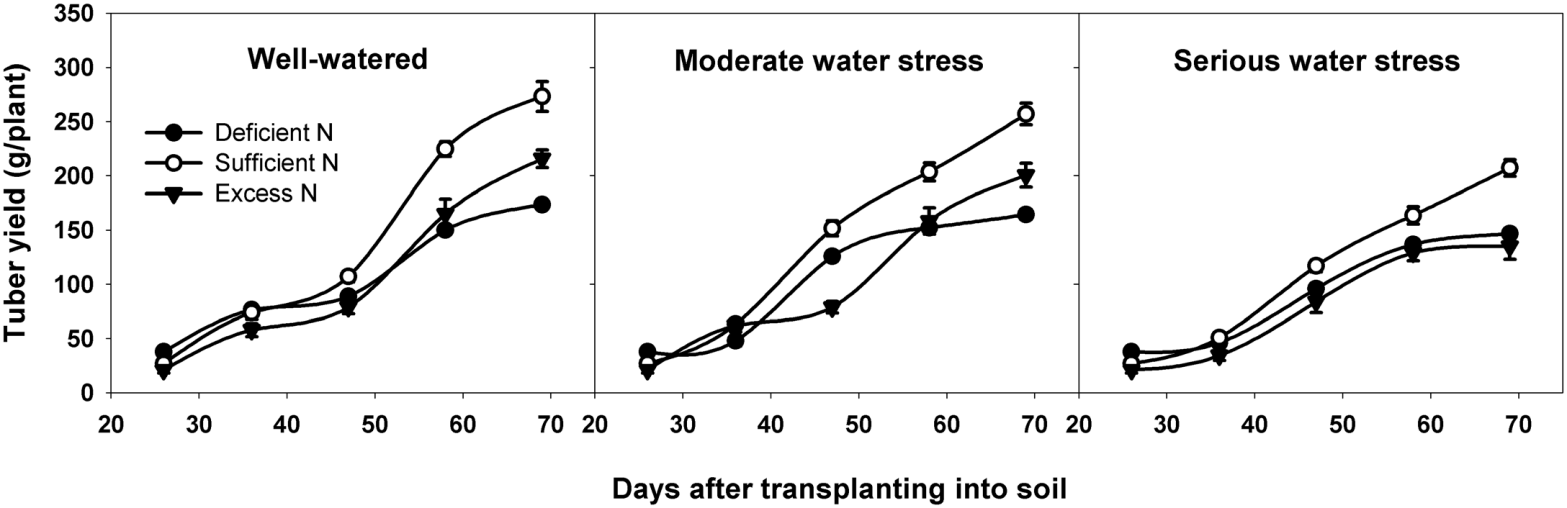
Tuber yield of potato at different DAT under different treatments. DAT represents days after transplanting into soil. All data are presented as the mean ± SE (n = 8).

There was a shift in potato tuber yield among different water and nitrogen levels (Fig 2). Water level, nitrogen level and water × nitrogen interaction all significantly influenced tuber weight at different harvest days (Table 2: Water *p* < 0.001 at different DAT; Nitrogen *p* = 0.018 at 36 DAT, *p* < 0.001 at 47, 58, 69 DAT; Water × Nitrogen *p* = 0.014, 0.001, 0.016, 0.028 at 36, 47, 58, 69 DAT, respectively). Under well-watered conditions, tuber yield was higher under sufficient nitrogen than under deficient and excess nitrogen. Under sufficient nitrogen, tuber yield was higher under well-watered than under moderate water stress and serious water stress. Thus, it seems that well-watered and sufficient nitrogen are beneficial to tuber yield, while water stress, deficient nitrogen and excess nitrogen all decrease tuber yield.

**Table 2:** ANOVA results for comparison of tuber yield and other relevant parameters at different DAT. DAT represents days after transplanting into soil.

	DAT		36			47			58			69		
		df	Mean square	*F*	*p*	Mean square	*F*	*p*	Mean square	*F*	*p*	Mean square	*F*	*p*
	Water	2	3923.3	22.3	0.000	4733.6	17.2	0.000	8431.1	25.2	0.000	21947.6	34.9	0.000
Tuber yield	Nitrogen	2	753.8	4.3	0.018	12010.9	43.6	0.000	13946.3	41.7	0.000	45812.8	72.9	0.000
	Water × Nitrogen	4	599.0	3.4	0.014	1401.7	5.1	0.001	1114.3	3.3	0.016	1840.8	2.9	0.028
	Water	2				38.1	119.3	0.000				1.3	5.8	0.007
Net photosynthetic rate	Nitrogen	2				84.0	263.4	0.000				457.7	2046.7	0.000
	Water × Nitrogen	4				6.7	21.0	0.000				4.3	19.1	0.000
	Water	2	900317.9	9.2	0.000	2112278.4	31.2	0.000	1845894.7	27.0	0.000	1935973.8	21.2	0.000
Total leaf area	Nitrogen	2	1996382.5	20.3	0.000	5272631.1	77.8	0.000	7291696.2	106.5	0.000	21316809.7	233.3	0.000
	Water × Nitrogen	4	68962.1	0.7	0.594	243820.2	3.6	0.010	591691.7	8.6	0.000	523984.9	5.7	0.001
	Water	2	0.5	0.4	0.685	15.7	13.4	0.000	6.4	5.0	0.012	24.3	26.8	0.000
Aboveground biomass	Nitrogen	2	15.0	12.1	0.000	43.9	37.4	0.000	64.4	50.2	0.000	99.2	109.4	0.000
	Water × Nitrogen	4	0.4	0.3	0.878	2.4	2.0	0.110	2.5	2.0	0.118	6.8	7.5	0.000
	Water	2	1792.4	105.8	0.000	1407.4	66.6	0.000	1526.1	100.6	0.000	291.1	17.3	0.000
Non-structural reserves content in leaves	Nitrogen	2	115.2	6.8	0.007	217.3	10.3	0.001	1366.2	90.1	0.000	1557.8	92.7	0.000
	Water × Nitrogen	4	87.5	5.2	0.007	60.2	2.9	0.056	103.1	6.8	0.002	28.8	1.7	0.196
	Water	2	231.9	2.2	0.157	540.5	2.1	0.179	10367.7	58.4	0.000	46.1	1.1	0.362
Non-structural reserves content in underground stems	Nitrogen	2	996.3	9.6	0.005	751.4	2.9	0.105	2327.4	13.1	0.001	296.4	7.2	0.010
	Water × Nitrogen	4	738.7	7.1	0.006	1132.2	4.4	0.030	925.9	5.2	0.009	37.4	0.9	0.494
	Water	2	22.1	8.0	0.001	8.4	3.4	0.038	0.3	0.2	0.861	11.8	5.6	0.006
Tuber number per plant	Nitrogen	2	23.1	8.4	0.001	28.2	11.6	0.000	24.2	13.8	0.000	15.4	7.3	0.001
	Water × Nitrogen	4	4.6	1.7	0.166	2.3	0.9	0.453	0.6	0.4	0.841	3.4	1.6	0.185
	Water	2	17.9	1.3	0.272	157.1	10.1	0.000	329.4	5.8	0.005	97.4	1.8	0.177
Average tuber weight	Nitrogen	2	18.5	1.4	0.259	13.5	0.9	0.424	280.1	4.9	0.010	357.5	6.5	0.003
	Water × Nitrogen	4	16.5	1.2	0.307	5.2	0.3	0.852	79.5	1.4	0.244	123.3	2.3	0.073
	Water	2	24.2	13.4	0.000	6.9	3.1	0.083	12.3	39.7	0.000	2.4	1.2	0.345
Nitrogen content in leaves	Nitrogen	2	908.4	504.3	0.000	621.9	285.4	0.000	735.1	2363.2	0.000	1966.4	1012.6	0.000
	Water × Nitrogen	4	58.3	32.4	0.000	11.6	5.3	0.012	4.5	14.5	0.001	0.9	0.5	0.769
	Water	2	1.2	1.4	0.317	11.9	27.6	0.000	1.7	5.2	0.036	1.2	1.8	0.219
Nitrogen content in stems	Nitrogen	2	694.1	780.3	0.000	398.4	926.7	0.000	277.3	861.3	0.000	262.3	392.0	0.000
	Water × Nitrogen	4	4.9	5.5	0.033	1.3	3.0	0.098	1.2	3.6	0.057	2.0	3.1	0.075
	Water	2	0.4	5.4	0.029	0.4	3.8	0.065	4.4	25.1	0.000	1.4	37.8	0.000
Nitrogen content in tubers	Nitrogen	2	31.4	378.3	0.000	31.0	295.7	0.000	45.8	260.1	0.000	17.6	468.2	0.000
	Water × Nitrogen	4	2.3	27.7	0.000	0.8	8.0	0.005	3.2	18.4	0.000	2.2	59.7	0.000

df: degrees of freedom.

### Effects of different water and nitrogen levels on source capacity

**Fig 3:**
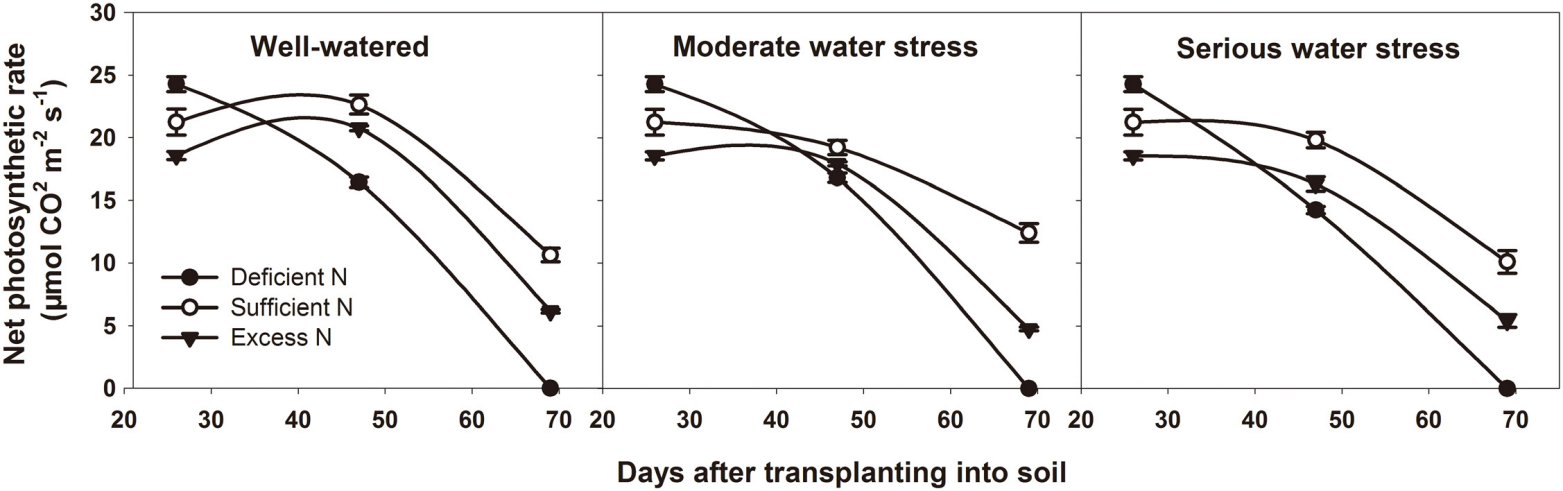
Net photosynthetic rate at different DAT under different treatments. DAT represents days after transplanting into soil. All data are presented as the mean ± SE (n = 5).

**Fig 4:**
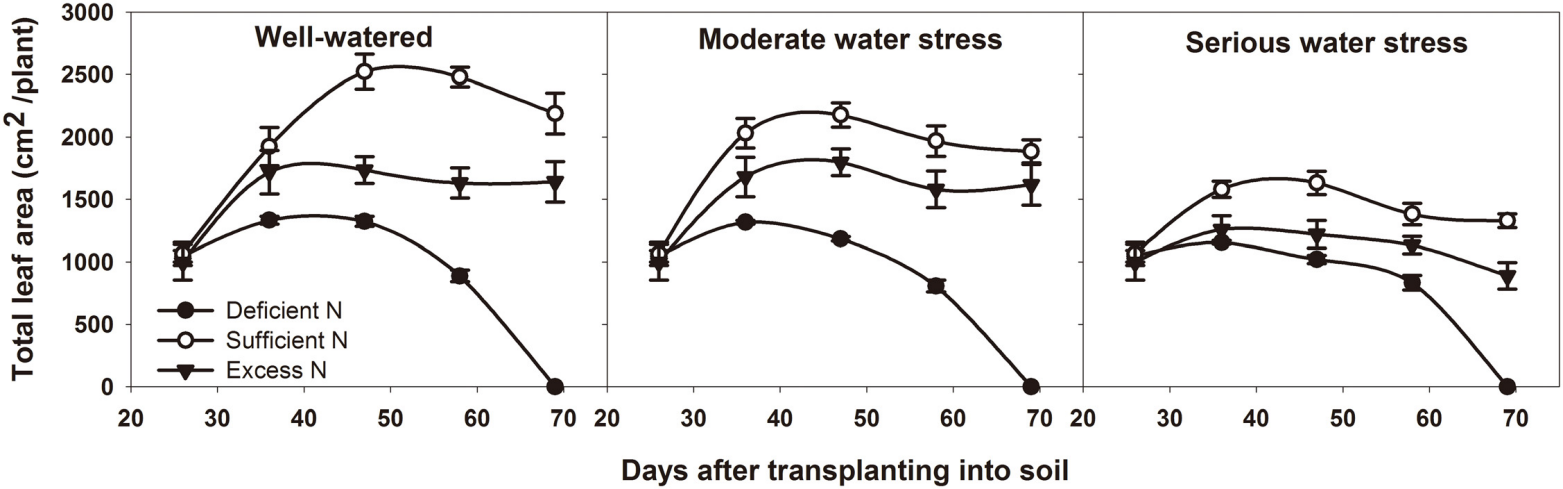
Total leaf area at different DAT under different treatments. DAT represents days after transplanting into soil. All data are presented as the mean ± SE (n = 8).

**Fig 5:**
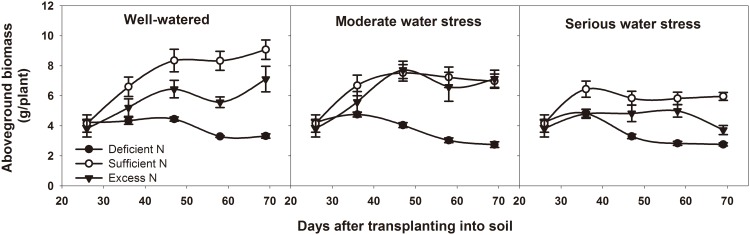
Aboveground biomass (leaves + aboveground stems) accumulated at different DAT under different treatments. DAT represents days after transplanting into soil. All data are presented as the mean ± SE (n = 5).

The growth of potato tubers depend on the assimilates supplied by the source. Source capacity affects yield directly. Our study used the net photosynthetic rate, total leaf area, aboveground biomass, non-structural reserves content in leaves and underground stems to assess the source capacity in potato. Table 3 showed that the tuber yield is correlated to the net photosynthetic rate, aboveground biomass and non-structural reserves content in underground stems during the tuber bulking stage. Among the three water levels, net photosynthetic rate, total leaf area and aboveground biomass were the highest under well-watered conditions and the lowest under serious water stress (Figs 3–5). Among the three nitrogen levels, net photosynthetic rate, total leaf area and aboveground biomass were the highest under sufficient nitrogen and the lowest under nitrogen-deficient conditions. During the tuber bulking stage, net photosynthetic rate and total leaf area decreased with DAT, but the decrease of total leaf area under sufficient nitrogen and excess nitrogen conditions were less than that under deficient nitrogen. It suggests that plants have a longer leaf life span and could produce more assimilates under sufficient nitrogen and excess nitrogen.

**Table 3:** Pearson correlation coefficients among tuber yield and some relevant parameters during the tuber bulking stage.

	Pn (μmol CO^2^ m^−2^ s^−1^)	TLA (cm^2^/plant)	AB (g/plant)	NRC_L_ (mg∙g^−1^ dry weight)	NRC_S_ (mg∙g^−1^ dry weight)	TN (/plant)	ATW (g)
Tuber yield (g/plant)	-0.626**	0.097	0.280**	-0.272	-0.445**	0.691**	0.931**

* significantly different at *p* ≤ 0.05;

** significantly different at *p* ≤ 0.01;

ns: not significant; Pn: Net photosynthetic rate; TLA: Total leaf area; AB: Aboveground biomass; NRC_L_: Non-structural reserves content in leaves; NRC_S_: Non-structural reserves content in underground stems; TN: Tuber number; ATW: Average tuber weight.

**Fig 6:**
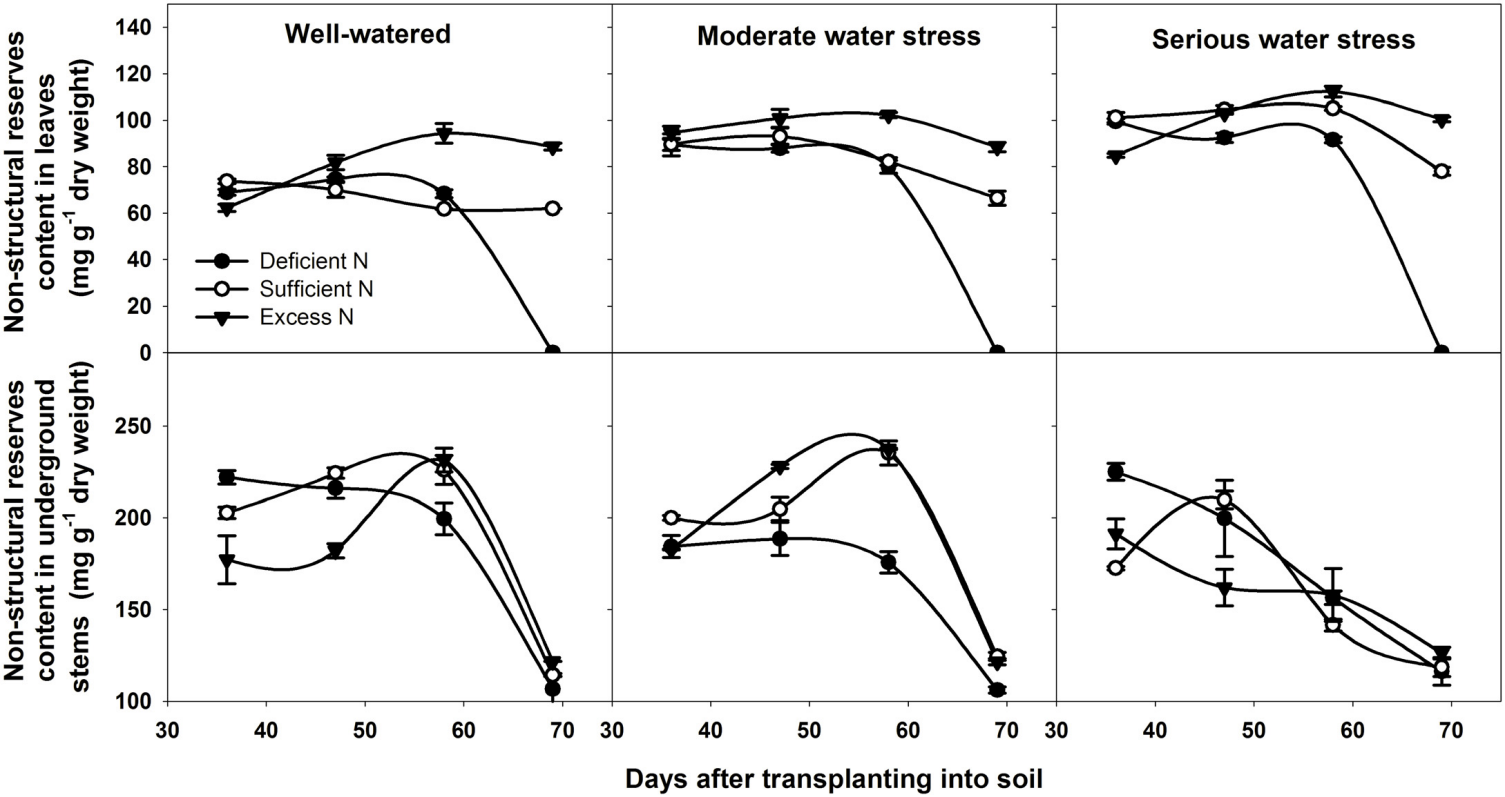
Non-structural reserves content in leaves and underground stems at different DAT under different treatments. DAT represents days after transplanting into soil. All data are presented as the mean ± SE (n = 3).

Fig 6 showed the non-structural reserves content of leaves and underground stems under different water and nitrogen levels. In leaves, the non-structural reserves content was higher under excess nitrogen than that under deficient and sufficient nitrogen in all three water levels. In underground stems, at 36 DAT, the non-structural reserves content was higher under deficient nitrogen compared to sufficient and excess nitrogen within well-watered and serious water stress. It may be due to the higher available nitrogen content in soil at 36 DAT under deficient nitrogen, since higher nitrogen application can reduce available nitrogen because of nitrogen immobilization [45]. At 69 DAT, under different water and nitrogen conditions, the non-structural reserves content in underground stems was relatively low and nearly equivalent. Meanwhile, plants under nitrogen-deficient conditions had no leaves because of senescence and defoliation, and consequently, they had no photosynthetic activity and non-structural reserves stored. This means that leaves under nitrogen-deficient conditions at 69 DAT could not supply assimilates to tubers. Thus, the low non-structural reserves content in the underground stem (about 120 mg∙g^−1^ dry weight) at 69 DAT may indicate an almost complete remobilization of stored carbon during tuber development, because stems need a certain amount of non-structural reserves to maintain its physiological activities. In wheat, Ruuska et al. reported that less than 50 mg∙g^−1^ dry weight of non-structural reserves content remaining in the stems represents full remobilization [46]. Therefore, our results suggest that the non-structural reserves are completely remobilized under all treatments, and there is no available non-structural reserves in the source of the underground stem at 69 DAT.

### Effects of different water and nitrogen levels on sink capacity

**Fig 7:**
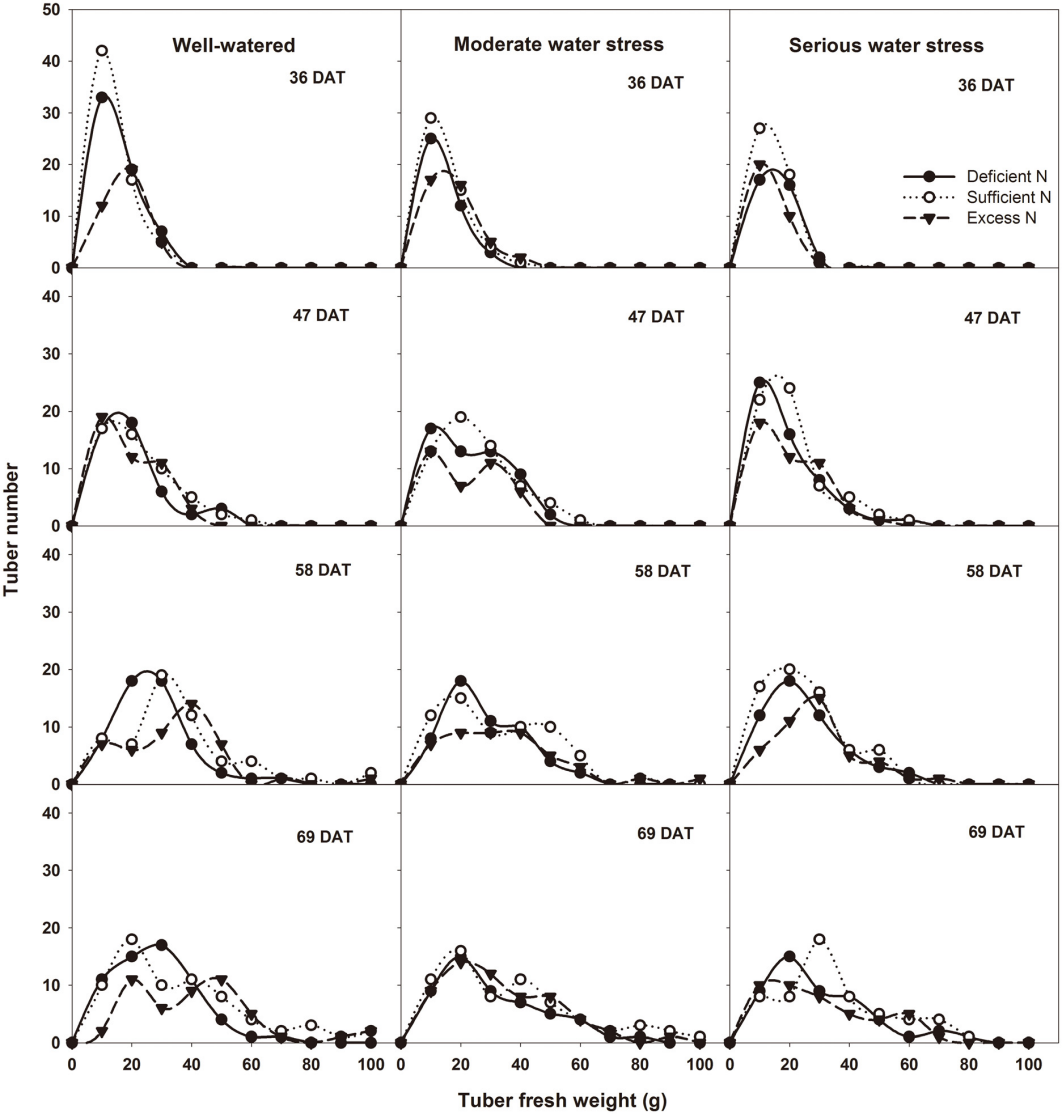
The distribution of number and weight of tubers at different DAT under different treatments. Abscissa represents fresh weight of each tuber, and ordinate represents the tuber number under this weight. DAT represents days after transplanting into soil, and each treatment includes 8 plants.

Sink capacity is the maximum space available for the accumulation of photoassimilates. In cereals, it is expressed as the number and size of grains [5]. Our study used the tuber number per plant and average tuber weight to access the sink capacity of potato. Fig 7 showed the distribution of weight and number of tubers. Among the three water levels, tuber number was largest under well-watered conditions and smallest under serious water stress. Among the three nitrogen levels, tuber number was larger under sufficient nitrogen than under deficient nitrogen and excess nitrogen. This suggests that well-watered and sufficient nitrogen are beneficial to tuber number, while water stress, deficient nitrogen and excess nitrogen are harmful. Under well-watered conditions, the tuber bulking rate was faster than under water stress. This resulted in a higher average tuber weight under well-watered conditions. Under deficient nitrogen and excess nitrogen, the tuber bulking rate was slower than under sufficient nitrogen conditions. This resulted in a lower average tuber weight under deficient nitrogen and excess nitrogen.

### Effects of different water and nitrogen levels on nitrogen content

**Fig 8:**
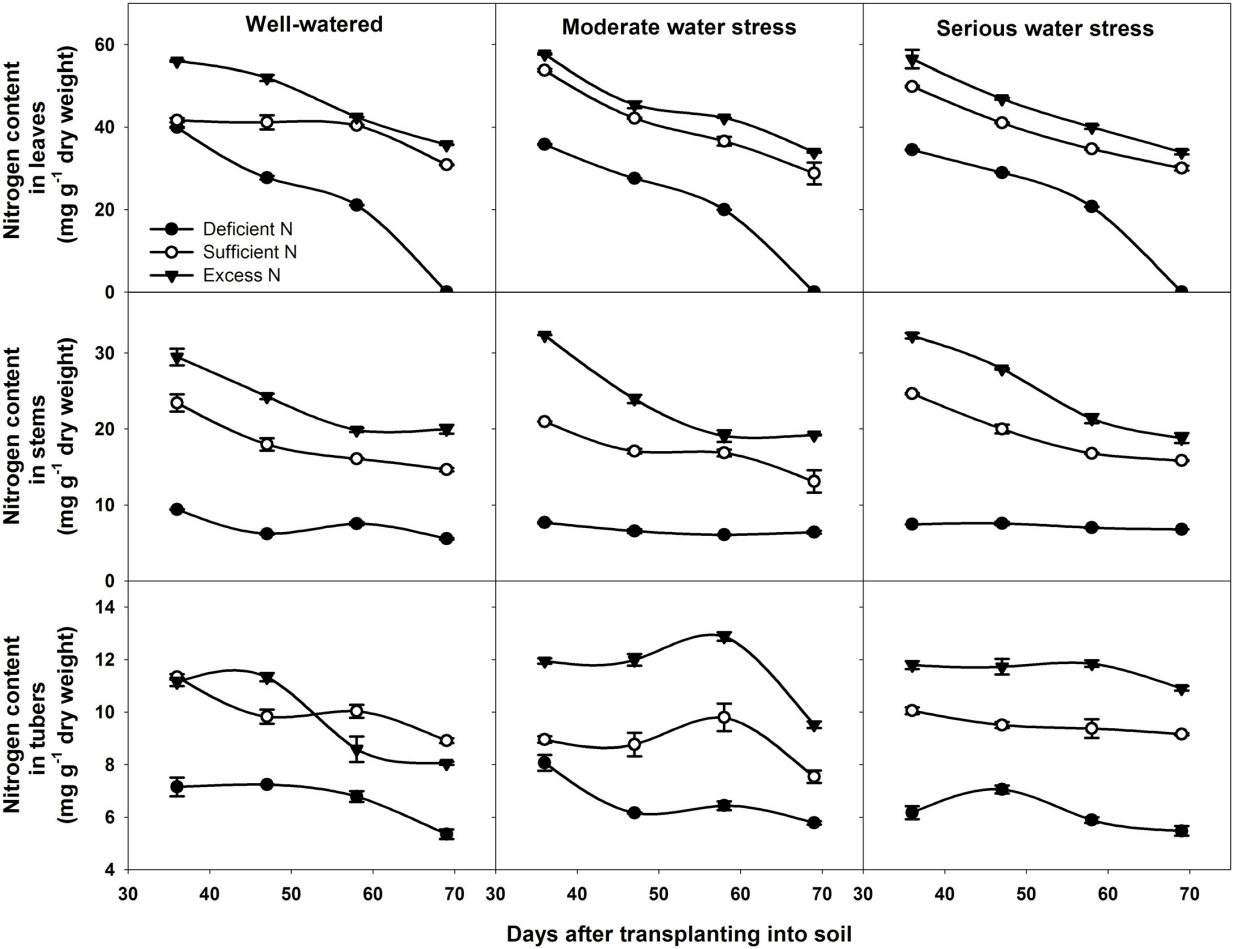
Nitrogen content in different organs at different DAT under different treatments. DAT represents days after transplanting into soil. All data are presented as the mean ± SE (n = 3).

Nitrogen content in different organs was significantly influenced by nitrogen supply (Table 2: in leaves *p* < 0.001 at different DAT; in stems *p* < 0.001 at different DAT; in tubers *p* < 0.001 at different DAT), and it increased with an increasing nitrogen level (Fig 8). Under excess nitrogen conditions, the nitrogen content in leaves, stems and tubers were all higher than it under deficient and sufficient nitrogen. For tuber bulking under all water conditions, nitrogen content of leaves decreased under all three nitrogen levels. In stems, the nitrogen content decreased under sufficient nitrogen and excess nitrogen conditions. Under nitrogen-deficient conditions, because of the low content throughout the experiment, nitrogen content changed little. In tubers, the nitrogen content decreased under the three nitrogen levels in well-watered and moderate water stress conditions. In serious water stress, nitrogen content changed little.

## Discussion

Source and sink are two key factors in determining cereal yield [47]. The source supplies the assimilates to the sink and the sink accepts and consumes these assimilates for its own growth or accumulates them for yield. Thus, the source-sink relationship resembles the supply-demand relationship [5]. In general, yield forming in cereals depends on two major sources: one source is the current canopy photosynthesis, and the other is the non-structural reserves stored in the canopy either pre- or post-anthesis [48–52]. Non-structural reserve stores provide the assimilate to grains during the dark period of the diurnal cycle and during the later grain filling stage, when the photosynthetic apparatus is senescing and the demands of sink growth exceed the source supply [53]. So the non-structural reserves remaining in the plant at maturity could indicate that there is an excessive source of assimilates in the plant during grain filling [54]. In potato, Zheng et al. reported that non-structural reserves stored in the stem also contribute to tuber growth [55]. Non-structural reserves are remobilized from underground stem first, because the underground stem is the closest to the tuber. Thus, the residual amount of stored reserves in underground stem at the end of the experiment (69 DAT) was used to determine the source and sink limitation in potato. Our results showed that, under normal growth conditions for tuber bulking (well-watered and sufficient nitrogen), net photosynthetic rate, total leaf area, leaf life span, tuber number per plant and average tuber weight were all the highest among all water and nitrogen levels (Figs 3–7). This means that source and sink capacity are both the highest under this condition, and thus result in the highest yield. Source capacity was compared with sink capacity under this condition, and we found that plant source capacity were very small at the end of the experiment, because the photosynthetic activity in leaves were low and non-structural reserves in underground stems were completely remobilized (Figs 3–6). While at this time, there was still a large potential capacity in the sink tubers. At 69 DAT, the maximum tuber number per plant was 12 and the minimum was 6; the maximum tuber weight was 96.78 g and the minimum weight was 1.52 g. This large variability in tuber number and weight suggests that the sink still has a large potential space to take in assimilates. This suggests that not enough assimilates were supplied by the source to meet the demands of growing sink tubers, and potato yield was limited mainly by the source capacity. Meanwhile, previous studies showed that elevated CO_2_ could increase leaf photosynthesis, tuber yield, and the nitrogen use efficiency in potato under sufficient nitrogen conditions [56–58]. The enhanced nitrogen use efficiency implies that CO_2_ enrichment may increase potato yield under deficient nitrogen, and potato yield may be source-limited under this condition. Our study also showed that under deficient nitrogen, the non-structural reserves content in leaves do not increase (Fig 6). Therefore, the low non-structural reserves content in the underground stems at 69 DAT may indicate an almost complete remobilization of stored reserves. The complete remobilization of underground stem reserves could be the representation of source limitation. In addition, Engels and Marschner determined the growth rates of individual tuber through reducing leaf area and tuber number, then suggested that tuber growth is limited by the source capacity during the phase of linear tuber bulking [59]. Sweetlove and Hill measured the transfer of ^14^C from CO_2_ to the tuber and leaf apoplastic sucrose concentration under different conditions, which also suggested that source metabolism dominates the control of source-to-sink carbon flux in tuberizing potato plants [60]. Therefore, we concluded that potato yield is more likely to be the source-limited under well-watered and sufficient nitrogen condition during the tuber bulking stage.

Water and nitrogen are two critical factors in potato production. Potato is sensitive to a water deficit [24]. Even slight water stress causes a reduction in leaf number and size, canopy radiation interception and photosynthesis, which consequently affects the tuber number, size and yield [61, 62]. Our results showed that, whether under moderate or serious water stress, net photosynthetic rate, total leaf area, leaf life span, tuber number per plant and average tuber weight are all lower than under well-watered conditions (Figs 3–7). This means that under water stress, source and sink capacities both are lower than those under well-watered conditions, which results in a lower yield. The lower source capacity was compared with the lower sink capacity, and we found that, at the end of the experiment, the source capacity were small since there were low photosynthetic activity in leaves and also no available non-structural reserves in underground stems. While at this time, the sink tuber still had a large potential capacity to take in assimilates since there was a large variability in tuber number and weight. At 69 DAT, the maximum tuber number per plant under water stress conditions was 10, while the minimum was 5; the maximum weight of the tubers was 92.14 g, while the minimum was 1.04 g. Therefore, this suggests that, although the source and sink capacities are both decreased by water stress, there is not enough source supply for sink growth, and tuber yield is more limited by the source than by the sink. In addition, this also suggests that the higher yield under well-watered conditions is mainly a result of the higher source capacity, because the sink tuber under well-watered conditions and the sink tuber under water stress conditions both had a large amount of potential space to take in assimilates at the end of the experiment.

Nitrogen is recognized as the most limiting nutrient for the potato crop, and potato yield is greatly affected by its availability [63, 64]. Nitrogen deficiency can reduce canopy growth and cause premature senescence, and thereby reduce yields. Excessive nitrogen can delay the linear tuber growth period for 7 to 10 days or slow tuber bulking in favor of vegetative growth, thereby reducing tuber yields [65, 66]. Our results showed that, under deficient nitrogen and excess nitrogen conditions, the net photosynthetic rate, total leaf area, leaf life span, tuber number per plant and average tuber weight are all lower than under sufficient nitrogen conditions (Figs 3–7). This means that under deficient nitrogen and excess nitrogen, source and sink capacities both are lower than under sufficient nitrogen, resulting in a lower yield. Source capacity was compared with sink capacity under these two conditions. The results showed that, plants with deficient nitrogen treatment had no source capacity at the end of the experiment. But at this time, the sink tuber still had a large potential capacity to take in assimilates because there was a large variability in tuber number and weight. Plants with excess nitrogen also had a large potential sink capacity at the end of the experiment. Meanwhile, the source capacity were small as showed that leaves had low photosynthetic activity and underground stems had no available non-structural reserves. Thus, it is suggested that although source and sink capacities are both lower under deficient nitrogen and excess nitrogen, there is an inadequate supply of assimilates to meet sink demands, and tuber yield is more likely to be the source-limited. In addition, it also suggests that the increased yield under sufficient nitrogen is mainly a result of a sufficient nitrogen increase in the potato source capacity, because the sink tuber under all three nitrogen levels had a large potential capacity at the end of the experiment. The decreased yield under excess nitrogen may be a result of a decreased source capacity under this condition. And this was consistent with the study of Evans in wheat, since he reported that CO_2_ assimilation rate increase with leaf nitrogen, but decline when leaf nitrogen exceeded a certain amount [67].

In agricultural production, influences of water and nitrogen on plant growth are not independent of each other, and they often interact [68–71]. Water stress reduces nitrogen uptake as a result of the decreased water uptake and transpiration rate [72]. On the other hand, a nitrogen deficit decreases root hydraulic conductivity, thereby affecting leaf water status and leaf growth [73]. In potato, our results showed that tuber yield is significantly affected by water and nitrogen interaction (Table 2: *p* = 0.014, 0.001, 0.016, 0.028 at 36, 47, 58, 69 DAT, respectively). Under water stress combined with deficient nitrogen or water stress combined with excess nitrogen conditions, the net photosynthetic rate, total leaf area, leaf life span, tuber number per plant and average tuber weight were all lower than under well-watered and sufficient nitrogen conditions (Figs 3–7). This means that under water stress combined with deficient nitrogen or water stress combined with excess nitrogen, source and sink capacities are both lower than under well-watered and sufficient nitrogen conditions, which results in a lower yield. Source capacity was compared with sink capacity under these conditions, and we found that, at the end of the experiment, the source capacity were small while the sink still had a large potential capacity. Therefore, this suggests that source supply is still not enough to meet the sink demand under these conditions, and potato yield is more limited by the source than by the sink. In cereals, previous studies showed that grain yield was sink-limited during grain filling and source-limited only when subjected to extremely severe stress [52, 54, 74]. Thus, our results suggest that the source-sink relationship in potato is different from that in cereals, and that potato yield is more likely to be the source-limited under all water and nitrogen levels during the tuber bulking stage. In addition, the analysis of variance showed that net photosynthetic rate, total leaf area, aboveground biomass and non-structural reserves content in the underground stem were all significantly influenced by water and nitrogen interaction, while tuber number per plant and average tuber weight were not significantly influenced (Table 2: Net photosynthetic rate *p* < 0.001 at 47, 69 DAT; Total leaf area *p* = 0.010, 0.001 at 47, 69 DAT, *p* < 0.001 at 58 DAT; Aboveground biomass *p* < 0.001 at 69 DAT; Non-structural reserves content in underground stems *p* = 0.006, 0.030, 0.009 at 36, 47, 58 DAT; Tuber number per plant *p* > 0.05 at different DAT; Average tuber weight *p* > 0.05 at different DAT). These indicate that the interaction between water and nitrogen influences yield by affecting potato source capacity, and not by affecting sink capacity. Under conditions where water and nitrogen are combined, the net photosynthetic rate, total leaf area and leaf life span changed with the change in nitrogen content in leaves, while the tuber number and average tuber weight did not change with the change in nitrogen content in tubers (Fig 8). This also suggests that water and nitrogen interaction influence source capacity, and do not influence sink capacity. Thus, the higher tuber yield under well-watered and sufficient nitrogen conditions compared with serious water stress and deficient nitrogen conditions is a result of the higher net photosynthetic rate, total leaf area and leaf life span (Figs 2–5). This means that well-watered combined with sufficient nitrogen conditions increases yield through increasing the source capacity. Therefore, this suggests that an increase in the net photosynthetic rate, total leaf area and leaf life span increases source capacity, which is crucial for potato yield improvement.

In summary, understanding the source-sink relationship in potato is important for improving potato yield. Under all water and nitrogen levels, the small source capacity and the large potential capacity of sink tubers at the end of the experiment suggest that not enough assimilates were supplied by the source to meet the demands of sink growth. Thus, we concluded that, unlike cereals, potato yield is more likely to be the source-limited during the tuber bulking stage. Water and nitrogen are two key factors in potato production. Our results showed that water level, nitrogen level and the interaction between water and nitrogen influence potato yield mainly through affecting the source capacity of potato. Well-watered, sufficient nitrogen and well-watered combined with sufficient nitrogen increased potato yield by enhancing the source capacity. Therefore, this suggests that an increase in the net photosynthetic rate, total leaf area and leaf life span to increase source capacity is crucial to improve potato yield. However, this experiment only used one cultivar, and additional studies using other cultivars are required to verify our conclusions.

## Supporting Information

S1 Dataset S1 Dataset contains data on soil water content, tuber yield, aboveground biomass, net photosynthetic rate, total leaf area, non-structural reserves content in leaves and underground stems, each tuber weight per treatment and nitrogen content in leaves, stems and tubers. (XLSX)
